# Transmission of yellow fever vaccine virus from breast feeding mothers to their infants: reporting of yellow fever virus (YFV) RNA detection in milk specimens

**DOI:** 10.12688/f1000research.74576.2

**Published:** 2022-03-15

**Authors:** Tarteel Hassan, Razan A. Bashir, Dina N. Abdelrahman, Hassan Madni, Abdel Rahim M El Hussein, Isam M. Elkidir, Khalid A. Enan

**Affiliations:** 1 Virology, Central Laboratory, Khartoum, Khartoum, Sudan; 2 Department of Microbiology and Parasitology, University of Khartoum, Khartoum, Khartoum, Sudan

**Keywords:** YFV, Vaccine, Milk, breast feeding mothers, Khartoum-Sudan

## Abstract

**Background:** Because of yellow fever’s serious impact on health, vaccination is the principal strategy to control the disease. Administration of the yellow fever vaccine to breastfeeding women should be before they complete 9 months post-delivery, in order to prevent transmission of the yellow fever vaccine virus to their infants through breast feeding. This study aimed to confirm whether the excretion of yellow fever vaccine virus is in milk of vaccinated breastfeeding mothers and to confirm the probable transmission to their infants through breast milk.

**Methods:** Samples were taken as follows: one serum specimen was taken 3-14 days after the date of the vaccination, and breast milk specimens were taken at four different time points between 3-4 days apart. Specimens were obtained from eight nursing mothers, who received the YVF vaccine (17DD). Mothers were asymptomatic before and after the vaccine administration but their infants developed symptoms after administration. Maternal serum samples were tested for YFV specific IgM antibodies through immuno-fluorescent assay (IFA). RNA was extracted from serum and breast milk specimens and YFV RNA screened using real-time polymerase chain reaction (RT-PCR).

**Results:** In total, five mothers (62.5%) were positive for YFV and two mothers (25%) had YFV RNA in serum. Among milk specimens, YFV RNA was detected during the four different mentioned collection times as follows (positive milk specimens/total milk specimens): 3/8 (37.5 %), 4/6 (66.6%) and 1/4(25%). RNA was completely undetectable in the last collection time.

**Conclusions:** YFV transmission from mothers to their babies through breast-feeding was highly probable indicated by the temporal relationship to mother’s YF vaccination.

## Introduction

The yellow fever virus is a mosquito-borne flavivirus that causes yellow fever, ^1^ a viral hemorrhagic fever that now occurs only in Africa, Central and South America, ^2^ but historically has had many wide outbreaks in Europe and North America. ^2^ Approximately 200,000 cases of yellow fever occur annually; 90% of them in Africa. ^2^ Yellow fever severity ranges from self-limited fever illness to hemorrhagic syndrome and death. ^3^ Most yellow fever patients are asymptomatic, but 15% develop severe illness, ^4^ which appears as a systemic illness that affects the liver (jaundice and necrosis), renal system, and myocardial system resulting in hemorrhage and shock. ^5^ Among the 15% of patients that develop severe illness, the mortality rate is 20%–60%. ^4^ Reports show that yellow fever is responsible for 29,000 to 60,000 deaths in South America and Africa every year, and it’s the most severe form of mosquito-borne diseases in tropical areas. ^1^

Because yellow fever is a potentially fatal disease, vaccination is the principal strategy to control the disease. ^4^ An effective yellow fever 17DD vaccine was established in 1937, but there are still currently over 400 million unvaccinated people in the areas of high infection risk. ^1^

Live attenuated 17DD vaccine confers protection in more than 95% of recipients within a month after single dose vaccination. Its protection is attributed to both innate and adaptive immunity through production of neutralizing antibodies directly against the envelope protein. 17DD vaccine administration provides immunity for at least 10 years and probably can extend to give life-long immunity. ^6^

The YFV vaccine can cause adverse side effects after its administration that range from mild to severe. The mild effects are headache, myalgia, and pain at the injection site, while severe effects can include anaphylactic shock, neurological diseases, and viscerotropic disease. The YFV vaccine is recommended to be administered after 9 months of age and from 6 months in endemic areas. ^7^ Administration of the vaccine to breastfeeding women before 9 months post-delivery can transmit the yellow fever vaccine virus to their infants with high risk of neurological diseases. There are three case reports of confirmed transmission of the YFV vaccine strain from mothers to their infants through breastfeeding. ^8^^–^^10^ The first case reported that the mother received the yellow fever vaccine 15 days after her delivery, and she had symptoms of headache, malaise, and fever after 20-22 days. ^8^ The other two cases reported that the mothers received a yellow fever vaccine, and their infants had developed encephalitis 3-4 weeks later. ^7^^,^^9^^,^^10^

Many studies have reported the transmission and presence of other flaviviruses RNA in breast milk such as West Nile virus (WNV), Zika virus, dengue and chikungunya ^11^^–^^14^; however, no previous study has reported the detection of YVF RNA in breast milk samples from vaccinated breast-feeding mothers in Sudan. Therefore, this study aimed to detect the yellow fever virus in breast milk and serum samples from vaccinated breastfeeding women whose infants got yellow fever illness to confirm whether the yellow fever vaccine virus is excreted and transmitted through breast milk. The mothers received the vaccine during an intensive YF vaccination campaign that involved millions of people in Khartoum State.

## Methods

### Ethical considerations

The study approved by the ministry of health, Sudan (approval number 5688-2019). Verbal consent was taken from mothers due to some being unlettered and some afraid of providing written consent. This was deemed adequate and approved by the Ministry of health.

### Study design and population

This study involved YFV testing in serum and breast milk specimens from eight nursing mothers (aged from 20-33 years) from Khartoum, Sudan in November 2019. The mothers had received the YFV vaccine (17DD) (Bio-Manguinhos/Fiocruz) during the 2019 YFV vaccination program in Khartoum within 9 months of delivery. They were not showing any symptoms of YVF before they got the vaccines; however, their infants (ages from 45 days to 8 months) developed symptoms of fever, diarrhea, jaundice, vomiting, and/or skin rashes after one week from their mother received the vaccines. Participants were approached via telephone through reports provided to the Ministry of Health to follow-up individuals who experience complications after getting vaccinated.

### Sample collection

The samples were collected by Ministry of health medical staff from participants in different hospital vaccination points. Blood samples were collected from mothers in a plain blood container, then serum separated from the blood sample through centrifugation at 1100 rpm for 15 minutes using centrifuge (Hettich- ZENTRIFUGEN), then serum samples pipetted into clean Eppendorf tubes and stored at -80°c until their use. Milk specimens were collected in a clean glass jar by hand expression whilst the infant was nursing on the other breast and vice versa. The specimens from the breasts (right and left) were expressed into separate clean glass jars. Collection of milk specimens performed on four occasions: the first collection time was 3-14 days from the date of the vaccination. The three following collections were 3-4 days apart. Milk specimens were collected on all occasions, but serum samples were collected only in the first collection.

### Serology

Maternal serum samples were tested for YFV specific IgM antibodies using immuno-fluorescent assay (IFA) according to manufacturer instructions (Yellow fever virus IIFT (IgM), EUROIMMUN, Germany, catalogue number Fl 2665-1005 M).

### RNA extraction

RNA was extracted from serum and breast milk specimens using a commercial RNA extraction kit (QIAamp viral RNA mini kit) according to the manufacturer instructions (Qiagen viral RNA, Germany). The extracted RNA was stored at −80°C until use.

### Real-time polymerase chain reaction (PCR)

Detection of YFV RNA was performed using real-time PCR (Rotor 5 plex real-time PCR machine Qiagen, Germany). Commercial kit which developed to detect all YFV strains including vaccine strain (RealStar ^®^ Yellow Fever Virus RT-PCR Kit 1.0, Germany) was used according to manufactures protocol. The PCR program consisted of 55°C for 20 min, 95°C for 2 min, followed by 45 cycles consisting of 95°C for 15 sec, 55°C for 45 sec and 72°C for 15 sec.

### Statistical methods

**Figure 1.**
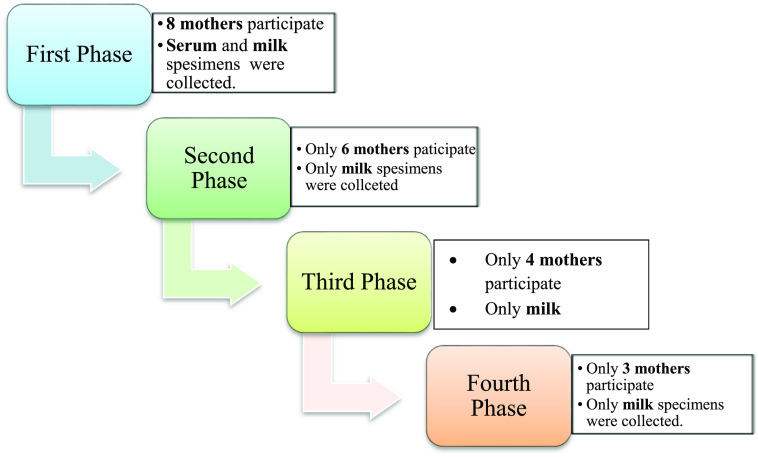
Participation per each phase of the study.

No statistical analysis was needed. Data from participants were documented in an Excel sheet containing data for each mother and their infants. Rotor 5 plex real-time PCR thermo cycler software used to create Figure 3 while Figures 1 and 4 were created using word.

## Results

The eight breast feeding mothers in Khartoum state were aged between 20 to 33 years old. ^15^ Participation per each phase of the study shown in Figure 1.

### Results of the first collection

**Figure 2.**
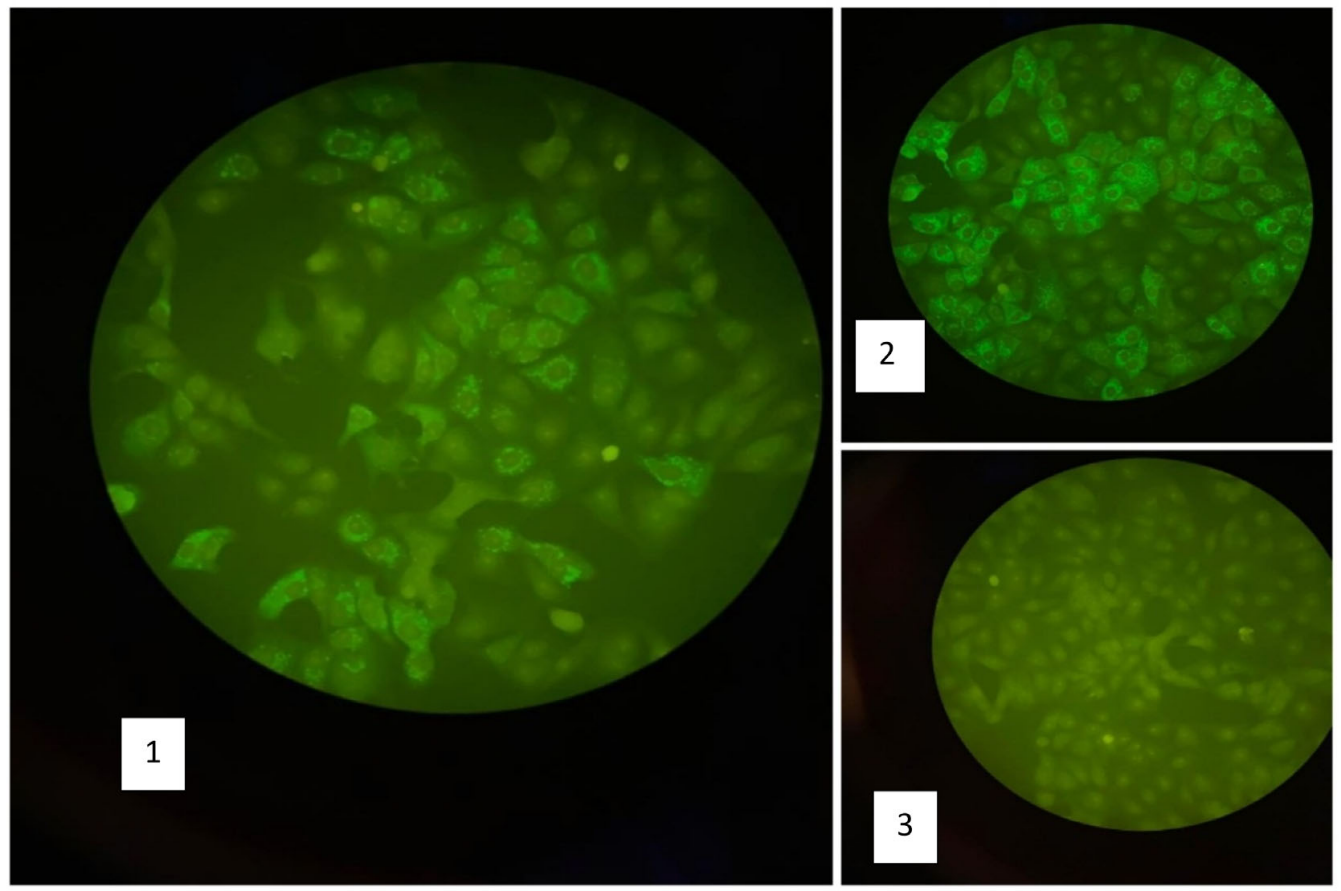
Result of anti-YFV IgM using IFA technique. (1) Positive sample for anti-YFV IgM, (2) Positive control for anti-YFV IgM, (3) negative control.

**Figure 3.**
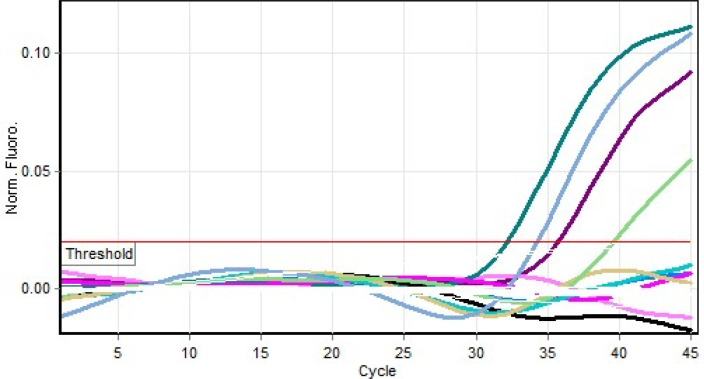
Real time polymerase chain reaction (PCR) result for yellow fever virus (YFV) positive milk specimens.

Five mothers (62.5%) showed IgM antibodies against YFV using IFA technique ( Figure 2). YFV RNA was detected by using real time PCR in 2 (25%) serum samples and 3 (37.5 %) in breast milk ( Table 1). Results of the PCR are shown in Figure 3.

**Table 1.** Results of serological and molecular detection of YFV.

Sample number	Date	Collection date	IFA-IgM	RT-PCR								
				serum	Milk							
					First		Second		Third		Fourth	
					Right	Left	Right	Left	Right	Left	Right	Left
**1**	3/11/2019	16/11/2019	**+**	**-**	**-**	**-**	+	+	-	+	-	-
**2**	4/11/2019	16/11/2019	**+**	**-**	**-**	**-**	+	+	-	-	-	-
**3**	6/11/2019	16/11/2019	**-**	**-**	**+**	**+**	+	-	-	-	-	-
**4**	5/11/2019	16/11/2019	**+**	**-**	**-**	**-**	-	-	NA *	NA *	NA *	NA *
**5**	12/11/2019	16/11/2019	**-**	**+**	**+**	**-**	+	-	-	-	NA *	NA *
**6**	12/11/2019	17/11/2019	**-**	**-**	**-**	**-**	-	-	NA *	NA *	NA *	NA *
**7**	5/11/2019	17/11/2019	**+**	**-**	**-**	**+**	NA *	NA *	NA *	NA *	NA *	NA *
**8**	6/11/2019	20/11/2019	**+**	**+**	**-**	**-**	NA *	NA *	NA *	NA *	NA *	NA *

PCR = polymerase chain reaction; YFV = yellow fever virus.

* Means no sample obtained.

### Results of the second collection

In this phase, only 6 samples were obtained, among them YFV RNA was demonstrated in 4 (66.6%) breast milk specimens ( Table 1).

### Results of the third collection

Four mothers were enrolled in this occasion. Only 1 (25%) of the milk samples was positive for YFV RNA ( Table 1).

### Results of the fourth collection

Milk specimens were obtained from 3 mothers, all specimens showed negative result for YFV RNAs ( Table 1). Minimal clearance of the viral RNA in milk was 11 day after vaccination and the maximum time needed for the clearance was 24 days after vaccine administration.

**Figure 4.**
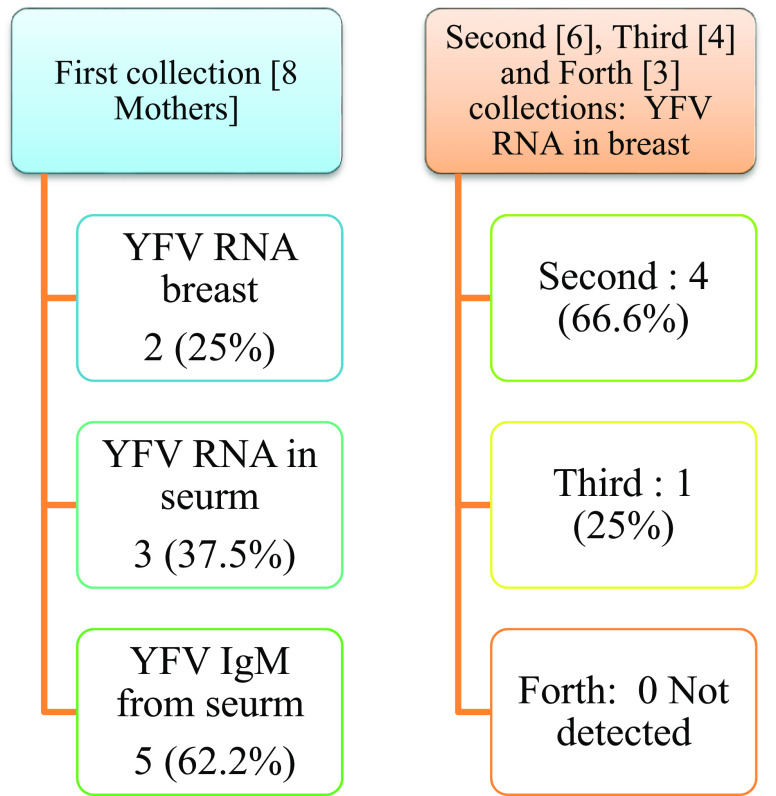
Result of yellow fever virus (YFV) detection among different collection dates.

Real-time PCR results showed in Figure 1 and results of yellow fever virus (YFV) detection among different collection dates showed in Figure 4.

## Discussion

According to the universally applied recommendations for YF vaccine, breast-feeding mothers and their infants who are aged less than 9 months should avoid YF vaccination. However, when breastfeeding mothers must travel to a yellow fever–endemic area, these women should be vaccinated, ^16^ and its recommended to temporary interrupt brestfeeding for at least 2 weeks after YFV vaccination. ^17^ In a CDC report, the prenatal transmission of vaccine strain was demonstrated without RNA detection in the milk samples, in other side our study prove that by RNA detection in milk samples. ^18^

This current study was the first study to confirm perinatal transmission of YFV, through detection of RNA in milk samples from vaccinated nursing mothers. In contrast a study by Eder Gatti (2020), reported that YFV RNA was not detected in serum and milk samples from vaccinated lactating mother. ^19^

Between 2009 and 2020, YFV RNA was usually detected in samples other than milk such as CSF and serum. On the other hand, in April 2009 the first probable case of YF vaccine strain transmission through breast milk was reported in a Brazilian infant; yellow fever-specific IgM antibodies were detected in serum and CSF and yellow fever vaccine strain viral RNA was found in CSF of the infant. However, no breast milk or maternal serum was collected for yellow fever virus testing.

On March 2011, another case report study showed a baby was developing encephalitis after his mother had received the YFV vaccine when he was 10 days old. ^10^ The authors claimed the probable transmission of the vaccine virus from the mother to her baby by detecting YF IgM in the infant’s serum and CSF. The clinical presentation, temporal relationship to the mother’s vaccination, and lack of other alternative pathogens were also strongly supportive of acute central nervous system infection with the vaccine strain of yellow fever. ^10^

Yet another study reported on detection of YFV IgM in a 38 days-old infant’s serum and CSF who was exclusively being breast-fed. The baby suffered from meningoencephalitis 3 to 4 weeks after the YFV vaccine administration to his mother. ^10^^,^^20^ The baby was discharged after the convulsive crises were controlled.

Many studies have reported on the detection of flaviviruses RNA (Zika, West Nile, dengue, and chikungunya) in human milk. ^7^ A study in 2017 described detection of viral RNA in serum and milk of three symptomatic breast-feeding mothers who were infected with Zika virus. ^13^ And in another study WNV RNA and IgM antibodies were detected in breast milk samples from mothers whose babies developed West Nile virus. ^14^ In one case of vertical transmission of dengue infection, the RNA of the virus was detected in blood samples from a mother and her child as well as in the mother’s breast milk. ^11^ Furthermore, another study reported that chikungunya virus RNA was detected in serum, urine and milk samples of a breast-feeding mother at third day of symptoms onset. ^12^

According to the previous reports of Yellow fever, all 3 reported cases of yellow fever were engaged to the vaccine virus strain, and in all reported cases RNA was not detected in breast milk. None of the reported cases was confirm detection of the virus in breast milk specimen, the only confirmed infection through viral RNA PCR detection was in cerebrospinal fluid of infant after his mother received vaccine, while in the other cases; serological detection of YFV IgM antibodies was performed in serum and cerebrospinal fluid. ^21^ Also Eder Gatti (2020), reported that YFV RNA was not detected in serum and milk samples from vaccinated lactating mother while specific IgM was detected. ^19^

This current study was the first study to confirm detection of YFV vaccine strain, through detection of RNA in milk samples from vaccinated nursing mothers. In contrast a case study reported by Ana Freitas (2020), showed detection of wild type YFV RNA genome in breast milk specimens from the mother. ^21^ Through searching it was un-able to find a report showing YFV vaccine strain presence in milk as we report.

This study has some limitations; because objections by mothers, no samples were collected from the infants in order to exclude other causes of the observed illness and to rule out an asymptomatic transmission of the virus. Another limitation is that the detected YFV RNA must be sequenced to confirm the identity as 17DD vaccine strain.

## Conclusion

Despite limitations, this study proves that YFV transmission from mothers to their babies through breast-feeding was highly probable indicated by the temporal relationship to mother’s YF vaccination. This also represents the first report on the detection of YFV RNA in human milk after YF vaccination in Sudan.

## Data availability

### Underlying data

Figshare: Transmission of yellow fever vaccine virus from breast feeding mothers to their infants: reporting of yellow fever virus (YFV) RNA detection in milk specimens.

https://doi.org/10.6084/m9.figshare.17206640. ^15^

Data are available under the terms of the Creative Commons Attribution 4.0 International license (CC-BY 4.0).
