# Supplementary material for: Transmission of yellow fever vaccine virus from breast feeding mothers to their infants: reporting of yellow fever virus (YFV) RNA detection in milk specimens
Source: F1000Res. 2024 Feb 29;11:76. Originally published 2022 Jan 21. [Version 4] doi: 10.12688/f1000research.74576.4 (PMC10724650; doi:10.12688/f1000research.74576.4)
Supplement: Peer review history

# Reviewer report: Reviewer response for version 3

**DOI:** 10.5256/f1000research.134910.r224060

**Published:** 2023-12-15

**Authors:** Albert Koulman

**Affiliations:** 1 Core Metabolomics and Lipidomics Laboratory, Wellcome Trust-MRC Institute of Metabolic Science, Addenbrooke's Treatment Centre, University of Cambridge, Cambridge, UK

One of the limitations is the sometimes very low concentrations of RNA. It is clear that for some of the samples, the detection threshold is reached between 30 and 35 cycles, while for other samples it is between 35 and 40 cycles. This means that the RNA concentrations are very low, at a level that contamination might be an issue. It is also questionable if such low concentrations are biologically meaningful. It would be good if the authors could provide the number of cycles necessary to reach the threshold for each sample. Is there exact information on the day the samples were collected? Please provide this information in Table 1. Please also provide information on the stages of breastfeeding. At the moment there is only mention of “within 9 months of delivery”, please provide at least the number of months since delivery for each mother.

 If the serum is negative for the RT-PCR test and negative for the IgM assay, it becomes difficult to understand why or how the milk became positive.

 The authors should also elaborate on the limitation section. It is important to note that the lactation changes and that the amount and type of cells and material in the milk will change during the whole lactation period. It could be that in early lactation the change of viral RNA getting into the milk is higher than in the later stages of lactation. Also, highlight that the concentration of RNA needs to be taken into account when assessing any risks for the infant.

Is the work clearly and accurately presented and does it cite the current literature?

Yes

If applicable, is the statistical analysis and its interpretation appropriate?

Not applicable

Are all the source data underlying the results available to ensure full reproducibility?

Partly

Is the study design appropriate and is the work technically sound?

Partly

Are the conclusions drawn adequately supported by the results?

Partly

Are sufficient details of methods and analysis provided to allow replication by others?

Yes

Reviewer Expertise:

lactation research and analytical chemistry

I confirm that I have read this submission and believe that I have an appropriate level of expertise to confirm that it is of an acceptable scientific standard, however I have significant reservations, as outlined above.

# Reviewer report: Reviewer response for version 2

**DOI:** 10.5256/f1000research.122300.r129788

**Published:** 2022-05-20

**Authors:** Ralph Huits

**Affiliations:** 1 Department of Infectious Tropical diseases and Microbiology, IRCCS Ospedale Sacro Cuore Don Calabria, Negrar, Italy

This manuscript reports on the testing of maternal serum for YFV specific IgM antibodies and YFV RNA, and serial breast milk samples for detectable YFV RNA, in eight nursing mothers, whose infants developed symptoms after the mothers received YVF vaccine. The authors found, for the first time, YFV RNA in breastmilk in five out of eight mothers 4-24 days after receiving 17DD vaccine. The detectable shedding of YFV (probably vaccine strain) in breast milk is the main message of this manuscript. This is an important finding that contributes to the evidence base for current YFV vaccination practices.

 Major comments:

 How were the mothers selected? It appears they were examined because their infants fell ill. This should be stated unambiguously, as it may underestimate the YFV RNA detection (and transmissibility) in breast milk.

 Strictly speaking, transmission was not confirmed, because no diagnostic data was presented for the infants.

 Minor comments:

 Introduction: (End of 1 ^st^ paragraph): 'the most severe form (?)', suggest rephrasing, e.g., ‘…, making it the most severe of mosquito-borne diseases.'

 The section on published case reports should include ref. 18.

 (Also note: ref. 9 = ref. 20, cited twice)

 Fig. 1: check spelling

 Results section: list the percentages after the nouns (e.g., 2 serum samples (25%)); In reporting the PCR results obtained in the second, third and fourth collections of breast milk, I would favour expressing the positive results as a fraction with the number of patients tested per timepoint in the denominator (e.g., 3/8, 4/6, etc.), rather than percentages that (falsely) lead back to the original number of patients (n=8).

 Table 1: Column headings: ‘Sample number’ should be ‘patient number’; ‘Date’ should be date of YFV vaccination’; For clarity it is probably more relevant to list the number of days between vaccination and timing of first sample collection, instead of the columns 2 & 3. Do ‘Right’/ ‘Left’ column headings refer to the right/left mamma? Please explain in legend.

 Fig. 3: check spelling; data in ‘YFV RNA in breast milk’ and ‘… in serum’ are not consistent with Table 1.

 Discussion: It is hard to ascertain whether recommendations are ‘universally applied’; the statement needs a reference. The reference for a 2-week interruption of breastfeeding before YFV vaccination is expert opinion. Could the authors comment on this duration based on their findings? (Detectable RNA in breastmilk up to 24 days post vaccination?)

 The CDC report cited (ref. 18) does confirm detection of YFV vaccine in the infant’s CSF. I suggest rephrasing. I have 2 comments on the statement ‘This current study was the first…’: this study was not the first, see ref. 18 above and in the next paragraph of the text- ref. 18 was not a probable, but a confirmed case.

 For Eder Gatti (2020): their surname is Fernandes. (Repeating findings from this paper on p. 8 is redundant).

Is the work clearly and accurately presented and does it cite the current literature?

Partly

If applicable, is the statistical analysis and its interpretation appropriate?

Not applicable

Are all the source data underlying the results available to ensure full reproducibility?

No source data required

Is the study design appropriate and is the work technically sound?

Partly

Are the conclusions drawn adequately supported by the results?

Partly

Are sufficient details of methods and analysis provided to allow replication by others?

Yes

Reviewer Expertise:

Arbovirus infections, malaria, tropical medicine, diagnostics evaluation.

I confirm that I have read this submission and believe that I have an appropriate level of expertise to confirm that it is of an acceptable scientific standard, however I have significant reservations, as outlined above.

# Response

**Published:** 2022-06-08

**Authors:** Dina Naser

**Affiliations:** Central Laboratory, Sudan

Dear **Ralph Huits**,

**Major comment:**

 The selected mothers had received the YFV vaccine within 9 months of delivery. They were not showing any symptoms of YVF before they got the vaccines; however, their infants developed symptoms of fever, diarrhea, jaundice, vomiting, and/or skin rashes after one week from their mother received the vaccines.

 Transmission to infants was confirmed through that their mothers get vaccinated week before developing symptoms and also through the detected YFV RNA in milk specimens from these same mothers during the infant’s appeared symptoms.

**Minor comments:**

Result:

**Do ‘Right’/ ‘Left’ column headings refer to the right/left mamma?**

 Yes, milk samples collected from both breasts in order to get the most excreted YFV in milk and also to study if there is any relation or correlation between breast side and YFV shedding in milk.

 *Other reviewer edits are done in the new submitted version.*

# Response

**Published:** 2022-06-22

**Authors:** Dina Naser

**Affiliations:** Central Laboratory, Sudan

In addition, some changes have been made in table, these changes are topical to that addressed online by the reviewer.

# Reviewer report: Reviewer response for version 2

**DOI:** 10.5256/f1000research.122300.r127591

**Published:** 2022-03-22

**Authors:** Leo G. Visser

**Affiliations:** 1 Department of Infectious Diseases, Leiden Medical University Center, Leiden, The Netherlands

The authors have addressed the concerns raised in the peer review report appropriately.

Is the work clearly and accurately presented and does it cite the current literature?

Yes

If applicable, is the statistical analysis and its interpretation appropriate?

Not applicable

Are all the source data underlying the results available to ensure full reproducibility?

No source data required

Is the study design appropriate and is the work technically sound?

Partly

Are the conclusions drawn adequately supported by the results?

Partly

Are sufficient details of methods and analysis provided to allow replication by others?

Yes

Reviewer Expertise:

Vaccine immunology, alternative vaccination routes, yellow fever vaccine, rabies vaccine, mRNA 1273

I confirm that I have read this submission and believe that I have an appropriate level of expertise to confirm that it is of an acceptable scientific standard.

# Reviewer report: Reviewer response for version 1

**DOI:** 10.5256/f1000research.78349.r120678

**Published:** 2022-02-07

**Authors:** Leo G. Visser

**Affiliations:** 1 Department of Infectious Diseases, Leiden Medical University Center, Leiden, The Netherlands

The authors report for the first time the presence of YVF RNA in breast milk specimens of five out of eight woman, three to fourteen days after receiving 17DD during vaccination campaign in Khartoum, Sudan.

**Major comment**

 Although transmission of YFV-17DD to the neonate through breastfeeding has been demonstrated before, ^1^ attempts to isolate the vaccine strain from breast milk have been unsuccessful. ^2^

 On the other hand, wild YFV has been detected in breast milk before. ^3^ Although there were no reports of a yellow fever outbreak in Khartoum state in November 2019, final proof would require to demonstrate that the nucleotide sequence of the amplified PCR product in the breast milk was identical to 17DD yellow fever vaccine virus. This limitation should be addressed in the discussion.

**Minor comments**

*Introduction*:

 The line “Administration of the vaccine to breastfeeding women before 9 months post-delivery can transmit the yellow fever vaccine virus to their infants through breast milk.” is confusing: breastfeeding is a risk factor for transmission irrespective of time post-delivery. However, the risk of YFV-associated neurological disease after transmission may occur in infants less than 6-9 months of age. The sentence should be corrected accordingly.

*Method*:

 What were the inclusion criteria? How were these woman selected? What is the cut-off CT-value for q-PCR result to be considered positive? Was a negative control included to check for contamination? What was the positive control for the YFV PCR?

*Results*

 Presentation of the results in a single table would increase readability. The data provided in Figures 1 and 4, and Tables 1 and 2 could be compiled into one table (time and specimen in the upper row, and subjects in left column). Figure 2 is redundant.

 In Figure 3 it is not clear from which specimen the amplification curves are presented? Is this serum or breast milk? The CT-values of 32 - 40 indicate very low levels of viral RNA. Maybe the authors could provide some more information on the CT-values in serum and breast milk.

*Discussion*

 Could the authors elaborate on how many weeks breast feeding should be interrupted to prevent transmission to the infant?

 The authors should discuss on the lack of confirmation that the nucleotide sequence of the amplified PCR product in the breast milk was identical to 17DD yellow fever vaccine virus.

Is the work clearly and accurately presented and does it cite the current literature?

Yes

If applicable, is the statistical analysis and its interpretation appropriate?

Not applicable

Are all the source data underlying the results available to ensure full reproducibility?

No source data required

Is the study design appropriate and is the work technically sound?

Partly

Are the conclusions drawn adequately supported by the results?

Partly

Are sufficient details of methods and analysis provided to allow replication by others?

Yes

Reviewer Expertise:

Vaccine immunology, alternative vaccination routes, yellow fever vaccine, rabies vaccine, mRNA 1273

I confirm that I have read this submission and believe that I have an appropriate level of expertise to confirm that it is of an acceptable scientific standard, however I have significant reservations, as outlined above.

## References

[1] : Transmission of yellow fever vaccine virus through breast-feeding - Brazil, 2009. *MMWR Morb Mortal Wkly Rep* .2010;59(5) :130-2 20150888

[2] : The search for yellow fever virus vaccine in breast milk of inadvertently vaccinated women in Brazil. *Rev Inst Med Trop Sao Paulo* .2020;62: 10.1590/s1678-9946202062033 e33 10.1590/s1678-9946202062033 32491144 PMC7266614

[3] : Detection of Wild-type Yellow Fever Virus in Breast Milk. *Pediatr Infect Dis J* .39(1) : 10.1097/INF.0000000000002496 68-69 10.1097/INF.0000000000002496 31725551
